# Preharvest Spray Hexanal Formulation Enhances Postharvest Quality in ‘Honeycrisp’ Apples by Regulating Phospholipase D and Calcium Sensor Proteins Genes

**DOI:** 10.3390/plants10112332

**Published:** 2021-10-28

**Authors:** Karthika Sriskantharajah, Walid El Kayal, Murali Mohan Ayyanath, Praveen K. Saxena, Alan J. Sullivan, Gopinadhan Paliyath, Jayasankar Subramanian

**Affiliations:** 1 Department of Plant Agriculture, University of Guelph, 50 Stone Road E, Guelph, ON N1G 2W1, Canada; sriskank@uoguelph.ca (K.S.); ayyanath@uoguelph.ca (M.M.A.); psaxena@uoguelph.ca (P.K.S.); asulliva@uoguelph.ca (A.J.S.); gpaliyat@uoguelph.ca (G.P.); 2 Department of Plant Agriculture, University of Guelph-Vineland Station, 4890 Victoria, Vineland, ON L0R 2E0, Canada; we21@aub.edu.lb; 3 Faculty of Agricultural and Food Science, American University of Beirut, Riad El Solh, Beirut 11072020, Lebanon

**Keywords:** bitter pit, calcium sensor proteins, ethylene, Harvista^TM^, hexanal, phospholipase D, phytohormones

## Abstract

‘Honeycrisp’ (*Malus domestica* Borkh.), a premium applecultivar, is highly susceptible to bitter pit and decline in quality during long-term storage. In order to enhance the quality, an aqueous composition containing hexanal was applied as a preharvest spray. The effects of hexanal were assessed on the treated fruit and compared with Harvista^TM^ (a sprayable 1-Methylcyclopropene based commercial formulation) applied and control fruit under both cold (2.5 °C; four months) and cold after room temperature storage (20 °C; 14 days) conditions. Color, firmness, and total soluble solids (TSS) did not show a significant change in response to any treatment at harvest, while abscisic acid (ABA) significantly reduced and tryptophan increased in response to hexanal, compared to Harvista^TM^ and control. The treatment effects on quality traits were observed during storage. Both hexanal and Harvista^TM^ sprayed apples had higher TSS under both cold and room temperature storage. In addition, both sprays enhanced firmness at room temperature storage. However, the effects of sprays on other quality traits showed a different pattern. Apples sprayed with hexanal had lower phospholipase D enzyme (PLD) activity, lower incidence of bitter pit, and decreased expression of *MdPLDα1* compared to Harvista^TM^ and control. On the other hand, Harvista^TM^ treated fruit produced lower ethylene. Both sprays decreased the expression of *MdPLDα4, MdCaM2, MdCaM4* and *MdCML18* genes. Generally, PLD alpha has a direct role in promoting fruit senescence, whereas the calcium senor proteins (CaM/CMLs) may involve in fruit ripening process via calcium and ethylene interactions. Therefore, improved postharvest qualities, including the lower incidence of bitter pit in hexanal treated ‘Honeycrisp’, may be associated with lower membrane damage due to lower PLD enzyme activity and decreased expression of *MdPLDα1* and *MdPLDα4* genes throughout the storage period.

## 1. Introduction

‘Honeycrisp’, a premium apple variety (*Malus domestica* Borkh), is mainly produced for fresh market. Since the year 2000, the production area and volume have risen tremendously due to increasing consumer demands [1,2]. Even though ‘Honeycrisp’ can make a profitable venture, the variety is highly susceptible to several serious physiological problems in common cold storage. For example, storing apples in long-term common cold storage frequently results in declining quality traits such as soluble solids, juiciness, and flavor [3,4]. Further development of storage disorders, including bitter pit (BP), can cause up to 50% postharvest yield losses [3,5]. Storing apple in controlled atmospheric storage does not work well for ‘Honeycrisp’ due to the development of various storage disorders, including CO_2_ injury and soft scald development [5,6,7]. Preconditioning can reduce the risk of soft scald development [8], but conditioning exacerbates BP in an already susceptible variety [5]. 

Previous studies have suggested that depletion of free apoplastic Ca^2+^ can weaken plasma membrane structure and function [9], leading to cell death and the development of BP symptoms [10,11]. Likewise, deteriorative changes in the plasma membrane due to physiological breakdown reduces the fruit quality. Phospholipase D (PLD) is a key membrane degradation enzyme that acts on the phospholipids and initiates a cascade of catabolic events that leads to membrane deterioration [12]. It has been identified that increased phospholipid degradation was linked to the activation of PLD by external stimuli such as increased ethylene [13] and cytosolic calcium [12,14]. 

Plant hormone ethylene is a key regulator of climacteric fruit ripening [15]. Even though ethylene concentration in ‘Honeycrisp’ is relatively low and stable during ripening, compared to ‘McIntosh’, a rapidly softening variety [16], a burst of ethylene production during fruit ripening triggers a series of physiological changes, including losses in firmness and crispness [17]. 1-Methylcyclopropene (1-MCP), an ethylene receptor blocker, prevents ethylene binding to its receptors, thus regulating the tissue response to ethylene. The application of 1-MCP helped maintain acidity and reduce ethylene production, skin greasiness [18,19], and certain storage disorders [20,21] in apples. Tomato treated with 1-MCP showed a marked reduction in PLD transcripts and slowed ripening process [22]. Harvista^TM^ is an orchard spray containing 1-MCP as an active ingredient that helps control fruit drop, reduce ethylene production, and retain firmness in ‘Golden Delicious’ [23] and McIntosh apples [24]. In addition, Harvista^TM^ decreased stem end flesh browning in Gala apples [25]. In previous studies, a significant effect from Harvista^TM^ in ‘Honeycrisp’ was noticed in controlling fruit drop and delaying harvest, but little effects were observed in storage quality traits and disorders [26,27]. However, the effects of Harvista^TM^ vary with several application parameters, including concentration, rate, and storage temperature. Therefore, Harvista^TM^ may exert beneficial effects on the shelf life and quality of ‘Honeycrisp.’ 

Phytohormones, particularly abscisic acid (ABA), auxin, gibberellins (GA) cytokinin, jasmonic acid (JA) and brassinosteroids (BR), are also implicated in fruit ripening in climacteric fruit. ABA concentration in apples reaches a peak just before commercial harvest [28], and maximum endogenous ABA preceded ethylene burst in apples [29]. In general, ABA and gibberellins (GAs) are one pair of phytohormones, which antagonistically mediate several plant developmental processes, including fruit ripening. Likewise, JA accelerates fruit ripening [30], whereas BR suppresses fruit ripening and senescence. In plants, melatonin regulates diverse functions, including the acceleration of fruit ripening [31]. Tryptophan acts a precursor for wide range of metabolites production that are essential for plant and human health. The Climacteric fruit ripening process is a complex network of ethylene crosstalk with other phytohormones. Hence, the applications of longevity protection technologies are often effectively worked in combined applications [32]. Therefore, suitable technologies and methods to enhance the postharvest shelf life of apples are in high demand. The process of membrane degradation initiated by the action of PLD during ripening and senescence is also enhanced by cytosolic calcium (Ca^2+^) due to disruption of membrane compartmentalization and loss in function of plasma membrane ATPases [9,12,33]. Further, the increased cytosolic Ca^2+^ can be sensed by calcium sensor proteins such as calmodulins. The Ca^2+/^Calmodulin (CaM) complex activates phosphatidate phosphatase leading to downstream membrane deterioration cascade events [12,34]. Calmodulin is a ubiquitously present, well-characterized calcium sensor protein that has EF-hand motif/s to bind Ca^2+^ [35,36,37]. Li et al. [38] have identified four CaM and 58 CML proteins containing functional EF-hand motifs in apples. Hexanal, a naturally occurring C6 volatile aldehyde, is a strong inhibitor of PLD activity. Hexanal also decreased ethylene in ripening fruit such as mango [39] and banana [40] and downregulated ethylene biosynthesis genes in tomato [13] and apple [41]. It has been suggested that the application of hexanal as an aqueous formulation enhanced membrane stability through inhibiting PLD activity and thereby improved marketable qualities and shelf life of several fruit and vegetables [42,43].

We previously reported that preharvest spray hexanal formulation delayed fruit abscission in ‘Honeycrisp’ most likely by minimizing ABA through an ethylene-dependent mechanism [41]. Hexanal formulation also decreased storage disorder bitter pit [44]. However, there is no information on underlying mechanisms on how hexanal improves postharvest qualities in ‘Honeycrisp’ during long-term storage. Here, we hypothesized that hexanal enhances the quality of ‘Honeycrisp’ apples by improving membrane integrity by regulating PLD activity via minimizing ethylene production and downregulating genes encoding the PLD enzyme. Thus, the objectives of this study were to evaluate the changes in storage qualities, gene expression of PLD and calcium sensor proteins in ‘Honeycrisp’ through the pre-harvest application hexanal formulation and compare its effects with Harvista^TM^ (an ethylene receptor blocker) and control. 

## 2. Results 

### 2.1. Effect of Preharvest Spray on Quality Parameters and Phytohormones at Harvest

Changes in color intensity, quality and phytohormones are important indicators of maturity and quality of fresh apples. No significant differences in any measured quality traits (except color coordinate b* of the background color) were observed among the treatments at harvest (Table 1). However, significant changes in phytohormones levels were observed among the treatments (Table 1). Apple treated with Harvista^TM^ produced 25% significantly lower ethylene compared to control (*p* = 0.0113). Hexanal-treated apples, on the other hand, produced 18% and 38% less ABA than control (*p =* 0.0399) and Harvista^TM^ (*p <* 0.0001), respectively. The concentration of zeatin was significantly greater in both hexanal and Harvista^TM^ treatments than in control (*p* < 0.0001). Similarly, tryptophan level was about 3 and 1.5 times greater in hexanal-treated apple than in control (*p* = 0.0001) and Harvista^TM^ (*p* = 0.0341), respectively. We could not detect other metabolites such as JA, indole-3-acetic acid, SA, N-acetyl serotonin, tryptamine, benzylamino amine, and Z-iP using UPLC-MS (Waters limited, Mississauga, ON, Canada) in the fruit samples at harvest. Presumably, those metabolites are present below the detection limit of the UPLC-MS.

### 2.2. Effect of Preharvest Spray on Ethylene and Phospholipase D Enzyme at Cold Storage 

#### 2.2.1. Ethylene Production 

**Figure 1:**
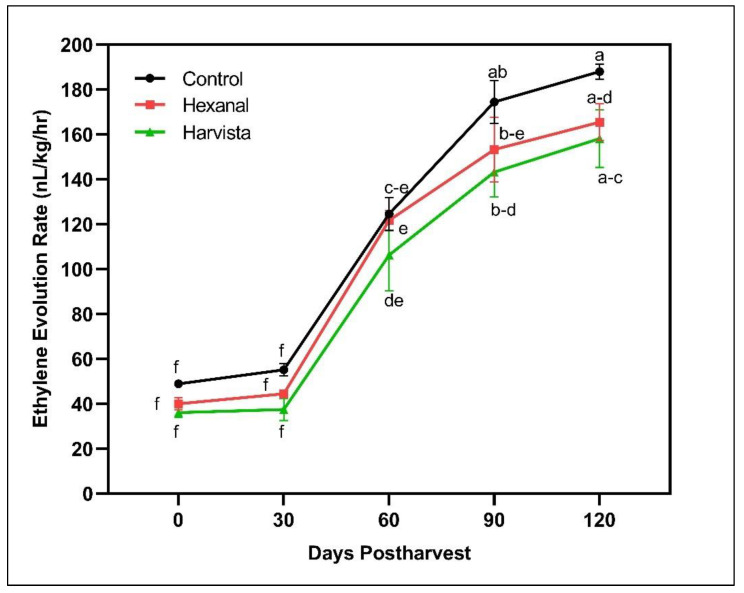
Effects of preharvest sprays hexanal and Harvista^TM^ on ethylene in ‘Honeycrisp’ apple throughout 120 days postharvest. Each value represents the least-squares means ± SE of eight fruit. LS-means with the same letter are not significantly different when comparing treatments with days postharvest based on the Tukey-Kramer test at α = 0.05.

Ethylene production consistently increased over time in all treatments (Figure 1), but on average, Harvista^TM^-treated fruit produced lower ethylene compared to control fruit (*p* = 0.0197) (Figure 1). On the other hand, ethylene production in hexanal-treated fruit did not significantly vary from Harvista^TM^ (*p* = 0.2097) or control (*p* = 0.0716). The rate of ethylene production from harvest to 90 days postharvest was higher in control (49–174 nL/kg/h), followed by hexanal (40–153 nL/kg/h) and Harvista^TM^ (36–143 nL/kg/h). The rate of increment in ethylene production revealed that preharvest sprays hexanal and Harvista^TM^ could reduce ethylene production by 12% and 18% after 120 days postharvest than control, respectively. 

#### 2.2.2. Phospholpase D (PLD) Enzyme Activity 

**Figure 2:**
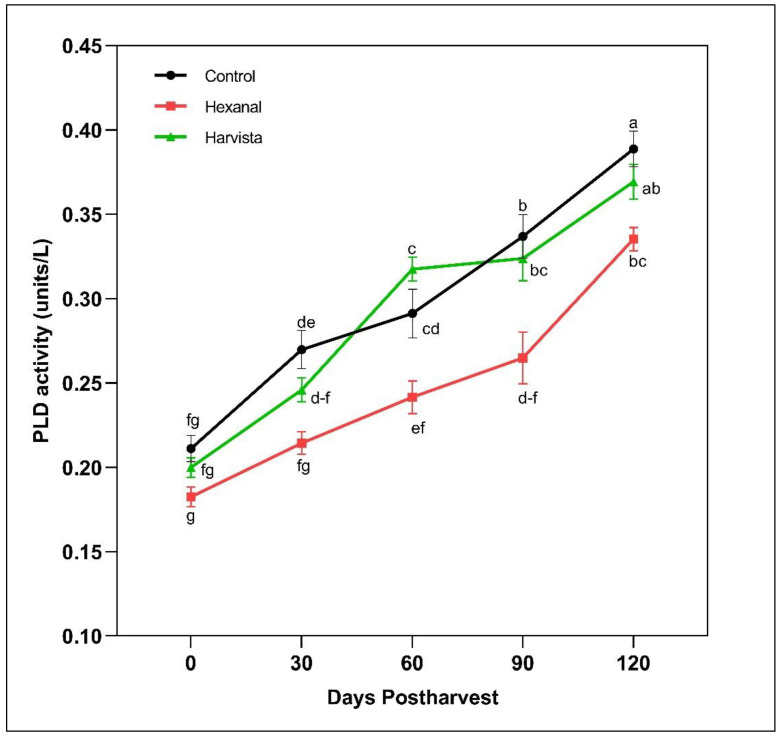
Effects of preharvest sprays hexanal and Harvista^TM^ on phospholipase D (PLD) activity in ‘Honeycrisp’ apple throughout 120 days postharvest. Each value represents the least-squares means ± SE of nine replicates. LS-means with the same letter are not significantly different when comparing treatments with days postharvest based on the Tukey-Kramer test at α = 0.05.

PLD enzyme activity increased throughout the storage in all treatments (Figure 2). As expected, PLD activity in hexanal-treated fruit was significantly lower than Harvista^TM^ (*p* = 0.0005) and control (*p* = 0.0002). Interestingly, a significant effect of hexanal treatment on PLD activity was maintained throughout storage compared to that of control. Hence, the PLD activity was consistently lower at all time points, showing a significant difference from control besides 30 days postharvest. Likewise, a significant difference between hexanal and Harvista^TM^ treatments was observed between 60 and 90 days postharvest, where hexanal maintained significantly lower PLD activity than Harvista^TM^. On the contrary, PLD activity in the Harvista^TM^ treated fruit fluctuated throughout the storage. These results showed that hexanal could inhibit the PLD activity by 19% compared to control at 120 days postharvest, while Harvista^TM^ can reduce only about 5% respective to control. 

### 2.3. Effects of Preharvest Spray on Bitter Pit (BP) Development 

**Figure 3:**
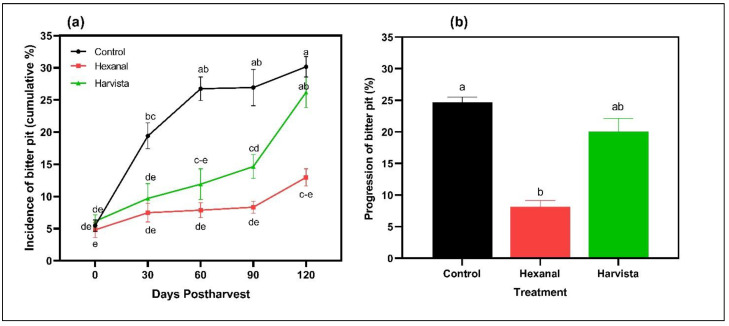
Effects of preharvest sprays hexanal and Harvista^TM^ on (**a**) incidence and (**b**) progression of bitter pit (BP) in ‘Honeycrisp’ apple throughout 120 days postharvest. Incidence of BP was calculated based on visual observation on present or absent of BP signs in the fruit. Progression of BP was calculated based on the difference in incidence of BP between 0 days postharvest and 120 days postharvest. Each value represents the least-squares means ± SE of three replications, and each replication had 14 fruit. LS-means with the same letters are not significantly different when comparing treatments with days postharvest based on the Tukey-Kramer test at α = 0.05.

The incidence of BP increased throughout the storage in all three treatments (Figure 3). However, the average value of the incidence of BP was significantly lower in hexanal treated fruit compared to control (*p* = 0.0002) and Harvista^TM^ (*p* = 0.0246). Further, the incidence of BP was remained largely unchanged throughout the postharvest in hexanal-treated fruit. However, on average, the incidence of BP did not statistically vary between control and Harvista^TM^ treated fruit (*p* = 0.2138). When the postharvest storage days increased, more fruit from the control group showed bitter pit signs. For instance, between 0 and 60 days postharvest, control fruit developed around 3.6- and 1.8-fold higher incidence of BP than hexanal and Harvista^TM^ treated fruit, respectively. Likewise, the progression of the bitter pit was significantly lower in hexanal treatment (*p* = 0.0046) compared to control. At the end of the 120 days of storage, about 86% of the hexanal-treated apples showed no signs of bitter pit compared to control (69%) and Harvista^TM^ (74%). These apples are considered marketable. 

### 2.4. Effect of Preharvest Sprays on Fruit Quality Traits during Cold Storage 

Fruit quality attributes such as color, firmness and TSS were measured throughout the postharvest to assess the effectiveness of treatments in improving/maintaining these fruit quality traits in ‘Honeycrisp.’ No significant differences in fruit firmness were observed across the treatments (Table 2). On average, TSS level was greater in hexanal (*p* = 0.0091), and Harvista^TM^ (*p* = 0.0195) treated apples compared to control. During the storage, TSS values fluctuated greatly in control fruit (12.89 to 13.55) whereas, in hexanal and Harvista^TM^-treated fruit, it was maintained between 13.40 to 13.65, 13.44 to 13.58, respectively (Table 2). Color parameters did not show a variation among the treatments at any sampling time (Supplementary Figure S1).

### 2.5. Expression Profiles of Genes Encoding PLD and Calcium Sensor Proteins 

**Figure 4:**
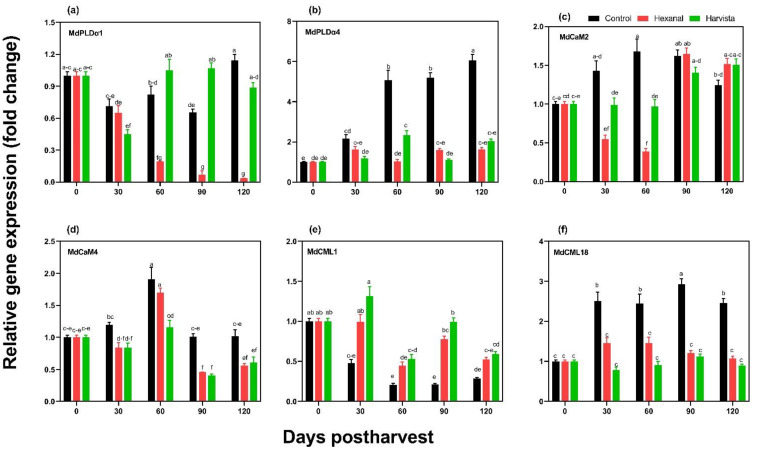
Effects of preharvest sprays hexanal and Harvista^TM^ on gene expression of two αPLD genes (**a**,**b**) and four calmodulin genes (**c**–**f**) in ‘Honeycrisp’ apple throughout 120 days postharvest. Transcript levels at all storage time points were expressed relative to their transcript level at 0 days postharvest. Each value represents the mean ± SE of three apples, with three replicates normalized against the housekeeping genes *MdAct* and *MdHis3*. Means with the different letters at the same storage time indicate significant differences among control, hexanal and Harvista^TM^ treatments based on the Tukey-Kramer test at α = 0.01.

Gene expression patterns of six genes, including two α-phospholipase D (MdPLDα1 and MdPLDα4) and four calmodulin genes (MdCaM2, MdCaM4, MdCML1, and MdCML18) (Supplementary Table S1), were quantified throughout the cold storage period. Transcript levels at all storage time points were expressed relative to their transcript level at harvest (0 days postharvest) (Figure 4). On average, the expression of both *MdPLDα1* and *MdPLDα4* were substantially lower in hexanal-treated fruit compared to control (*p* = 0.0001). Likewise, expression of *MdPLDα4* was lower in Harvista^TM^ treated fruit compared to control (*p* < 0.0001). The transcript levels of *MdPLDα1* in control and Harvista^TM^ treated fruit were relatively unchanged throughout the storage, while it progressively decreased in hexanal treated fruit and only trace levels of *MdPLDα1* transcripts could be detected beyond 90 days of storage (Figure 4a). However, the transcript of *MdPLDα4* in the control fruit was increased, while a significant reduction was observed in both hexanal and Harvista^TM^ treated fruit (Figure 4b). The expression of *MdCaM2*, *MdCaM4* and *MdCML18* was increased up to 60 days postharvest and remained unchanged or decreased in the control fruit. A treatment effect was observed in *MdCaM2*, *MdCaM4*, and *MdCML18* during this rising expression period (60 days postharvest) and beyond in *MdCaM4* and *MdCML18*. The transcript levels of the above genes were lower in the preharvest sprays than in control (Figure 4c,d,f). On the contrary, the transcript levels of the *MdCML1* were progressively decreased in the control fruit, while expression is significantly higher in both hexanal and Harvista^TM^ treated fruit (Figure 4e). 

### 2.6. Effects of Hexanal and Harvista^TM^ on Fruit Quality Traits at Room Temperature Storage

An experiment was conducted to study the effects of treatments on shelf life and quality of apples after removal from cold storage (2.5 °C) to room temperature) storage (~20 °C). The fruit was removed from cold storage after 30, 60 and 90 days postharvest and kept for another 14 days at room temperature. The quality measurements firmness, TSS and weight were recorded 7 and 14 days after placement at room temperature. Overall, quality traits did not vary between the 7th and 14th days of storage (Table 3). The treatment effects were observed on weight and TSS. Both hexanal and Harvista^TM^ treated fruit had higher TSS than control fruit at all sampling times (except at 14 days after removal from 60 days of cold storage), irrespective of days at cold and room temperature stored period (Table 3 and Table S2). Likewise, hexanal-treated fruit maintained significantly greater weight than control and Harvista^TM^ when the fruit was removed from 30- and 60-days cold storage (Table 3). Harvista^TM^ treated fruit had higher firmness than control when the fruit were removed from 30 (at both 7 and 14 days at room temperature) and 60 (7 days at room temperature) days of cold storage. Hexanal also maintained greater firmness than control only after removal of fruit from 30 days cold storage and kept for 14 days at room temperature.

## 3. Discussion

‘Honeycrisp’ is a highly valued apple variety. However, quality decline and development of storage disorder bitter pit cause up to 50% postharvest losses. In the present study, we evaluated the effects of preharvest spray hexanal formulation on postharvest qualities in ‘Honeycrisp’ apples, and the effects were compared with Harvista^TM^ (Harvista^TM^, AgroFresh Inc., Philadelpha, PA, USA) and control. 

The development of quality characteristics in ripening fruit involves several catabolic reactions that contribute to the organoleptic quality of the fruit [45]. However, accelerated catabolic breakdowns lead to quality decline and senescence process. Ethylene is a key regulatory factor in enhancing the activities of several enzymes involved in the catabolic reactions. Thus, blocking ethylene perception with chemicals such as Harvista^TM^ (an ethylene receptor blocker that contains active ingredient 1-MCP) is a technology that is currently in use for extending fruit retention and qualities in apples [23,27]. Likewise, metabolites channeling from degradative biochemical pathways into quality enhancing pathways can result in enhanced quality characteristics. Thus, by reducing membrane lipid degradation with hexanal, potentially enhanced shelf life of several fruit and vegetables, including raspberry [46], mango [39], banana [40], tomato [22] and bell pepper [43].

One of the significant findings of this study is the consistently improved soluble solids by hexanal and Harvista^TM^ in the cold (Table 2) and room temperature storage (Table 3). In addition, both hexanal and Harvista^TM^ have maintained firmness, specifically at room temperature (Table 3). Even though earlier studies [26,27] have mentioned that the Harvista^TM^ application has minimal effect on ‘Honeycrisp’ qualities, we could observe some positive effect of Harvista^TM^ during storage may be due to different time and rate of application. Generally, variations in firmness are poorly understood in ‘Honeycrisp’ due to the slow-softening nature of this variety [47]. Yet, higher firmness in the treated fruit may be associated with greater cell turgor and cell membrane integrity, as mentioned by Tong et al. [3] and Johnston et al. [17]. Consumer prefers the apple with greater firmness and crispness. Hence, improving firmness and taste would be an advantage for the cultivars such as ‘Honeycrisp’ as they are mainly cultivated for the fresh market. However, further experiments involving sensory panels, are required to show how the treatments affect sensory perception of consumers.

In addition to the above quality improvements, both preharvest sprays enhanced the tryptophan content at harvest. The increment was almost 1.5-fold higher in hexanal treatment compared to Harvista^TM^ (Table 1). Tryptophan is essential for protein synthesis and serves as precursors for a wide range of secondary metabolites such as indole acetic acid and indole alkaloids that are essential for plants and human nutrition and health [48]. Tryptophan also acts as a precursor for melatonin -a signaling molecule in plants and contributes to fruit ripening. The capacity of melatonin biosynthesis from tryptophan varies with the developmental stages [49]. For example, senescence induces more serotonin than melatonin. In the present study, no significant difference in melatonin among the three treatments was observed. ABA is another phytohormone that accelerates autocatalytic ethylene biosynthesis and thus accelerates the ripening process [28]. A significant reduction in ABA by hexanal at harvest might have delayed the ripening process in the treated fruit. Zeatin is a naturally occurring cytokinin, highly present in developing fruit than ripening fruit [50]. Both hexanal and harvista treated fruit contain more zeatin than control at harvest may have also associated with slow ripening. The mode of action of hexanal is so specific in maintaining membrane integrity by decreasing PLD enzyme activity [12]. As expected, PLD activity was substantially decreased in the hexanal treatment (Figure 2). Ethylene-induced gene expression is required for the production of the PLD enzyme [12]. Even though we could not notice a significant reduction in ethylene production in the hexanal treated fruit (Figure 1), a lowered expression of PLD genes (*MdPLDα1* and *MdPLDα4)* (Figure 4) might have contributed to lower the PLD turnover (Figure 2). Similar results were observed in the previous studies on raspberry [46], mango [39] and tomato [22], where hexanal substantially decreased the PLD activity and PLD genes, thus slowed down the ripening process and preserved the membrane. On the other hand, Harvista^TM^ treated fruit produced lower ethylene compared to hexanal. However, PLD activity fluctuated throughout the storage in the Harvista^TM^ treated fruit, suggesting that both orchard sprays have a different mode of action in regulating quality traits at harvest and during storage. 

With the progression of ripening and senescence, cytosolic calcium level rises due to several reasons, including increased ethylene production, progressive membrane degradation and inactivating calcium protons pumps [12]. Such Ca^2+^ can be sensed by cytoplasm localized calcium sensor proteins such as calmodulins (CaM) [35,51]. In tomatoes, CaM expression, especially *SlCaM2,* was upregulated by ethylene [52]. Similarly, in papaya set of *CaM/CML* expression were upregulated by ethephon but downregulated by 1-MCP during storage [53], indicating that the expression of *CaM/CML* is regulated by ethylene. Moreover, CaM/Ca^2+^ complex increases the activity of the phosphatidate phosphatase enzyme and thus accelerates the downstream membrane degradation process [12]. In the present study, the expressions of three (*MdCaM2, MdCaM4, MdCML18*) calmodulin protein genes were significantly lower in the preharvest sprayed fruit compared to control (Figure 4). The lower expression of CaMs during storage in sprayed fruit may partly support the fact that the cytosolic calcium rises in the sprayed fruit may be lower than the control fruit. 

The intact membrane acts as a barrier for preventing disorders, especially during long-term storage, as microcracking and softening of the epicuticular wax layer facilitate the development and progression of physiological disorders [54]. Storage disorder BP is characterized by dark deepening depressions that originated in the outer cortical cells below the skin of the apple as a result of cell membrane collapse and the death of localized clusters of cells [55,56]. In our study, hexanal treated fruit showed lower incidence and progression of the BP than control and Harvista^TM^ treated fruit. The lower incidence and progression of BP in the hexanal-treated fruit could be associated with lower cell membrane damage due to low PLD activity and decreased expression of *MdPLDα1* and *MdPLDα4* genes. The decreased expression of calcium bound-calmodulin protein genes such as *MdCaM2*, *MdCaM4* and *MdCML18* indicates controlled cytosolic calcium rises throughout the ripening. Hence, this is an indication of the lower incidence of BP in the hexanal-treated fruit.

In conclusion, our present study demonstrates the crucial role of preharvest hexanal spray in improving fruit quality traits during the long-term storage of the ‘Honeycrisp’ apple. The effects of hexanal and Harvista^TM^ were comparable at harvest as well as during storage. Both preharvest sprays have greatly influenced on hormone and metabolites than quality traits at harvest. However, both sprays enhanced the solid soluble content under both cold and room temperature storage conditions. Likewise, firmness was also maintained at room temperature storage. However, the effects of both sprays are different in maintaining some other quality traits under cold storage. Hexanal substantially reduced PLD activity, the incidence of BP, and *MdPLDα1* gene expression compared to Harvista^TM^ and control. Whereas Harvista^TM^ substantially reduced ethylene production. At the same time, both hexanal and Harvista^TM^ decreased the expression of *MdPLDα4, MdCaM2, MdCaM4,* and *MdCML18*. The mechanism of improved fruit qualities specifically the lower incidence of bitter pit by hexanal in ‘Honeycrisp’ is partly through inhibiting PLD activity and downregulating *MdPLDα1, MdPLDα4* expressions. Thus, hexanal promises to be a great technology to enhance the fruit qualities, marketability, and consumer appeal in the ‘Honeycrisp’ apple, given that this cultivar is categorized as susceptible to postharvest disorder BP. 

## 4. Materials and Methods

### 4.1. Experimental Location and Treatments

Fruit was harvested from 60, uniform, nine-year-old ‘Honeycrisp’ apple trees grown in a commercial orchard located within the Niagara region of Ontario, Canada (43°08′53.7″ N, 79°29′50.2″ W). The ‘Honeycrisp’ trees have ‘Mark 9’ (M.9) as their rootstock, and the average height of the canopy was about 3 m. The trees were supported by a trellis system and drip irrigation. The orchard grew apples for a specialty market that required a larger fruit size and deeper color.

An aqueous composition containing hexanal at a concentration of 0.02% (*v/v*) in the final spray was prepared as described in Kumar et al. [57]. In this case, 20 apple trees were subjected to two preharvest sprays of hexanal approximately 30 and 15 days before the commercial harvest (26 September 2019). A custom-built pressurized sprayer (Rittenhouse sprayers, St. Catharines, ON, Canada) was used for applying the hexanal solution at a rate of 1 L per tree to ensure that fruit was covered to the point of dripping with the treatment. Fruit was also picked from 20 ‘Honeycrisp’ trees which were sprayed with Harvista^TM^. Harvista^TM^ solution was prepared according to the manufactured protocol (12 lb/acre Harvista^TM^ mixed with 132 L/acre water) and applied seven days before the harvest using a commercial sprayer, Hol spraying system-CF series sprayer (Trailed sprayer, H.S.S./CG1000, Meteran, The Netherlands). The next group of 20 trees were not sprayed with any solution (control group). Three buffer rows and 10 untreated trees were maintained between the treatments to avoid spray contamination. 

### 4.2. Storage Studies 

Fruit that are uniform in size, similar maturity and without any defects were harvested, sorted, and packed into commercial boxes with liners accommodating 42 fruit per box. The boxes were immediately transported to a cold storage facility and stored at 2.5 °C (95%, relative humidity) for the next 120 days. Fruit standard quality parameters such as color, firmness and total soluble solids were assessed monthly. 

For the room temperature storage experiment, at the end of every 30 days of cold storage, 10 randomly selected fruit from each treatment were kept at room temperature (~20 °C) for another 14 days to assess the shelf life and quality changes of the fruit. Same fruit standard quality traits were measured at 7th and 14th days after the placement in the room temperature. 

### 4.3. Standard Quality Assessment during Storage

Two randomly selected fruit from each replication (box) representing eight fruit per treatment were used for the analysis. Blush and background colors were taken using a chromameter (CR-400, Konica Minolta Sensing Americas Inc., NJ, USA) according to the CIE Lab system readings (L—brightness, a—red/green and b—yellow/blue) values [58]. Chroma, a measure of color clarity (a^2^ + b^2^)^1/2^, and Hue angle ($\tan^{-1}\left(\frac{b}{a}\right)$ were calculated using the software available at http://www.easyrgb.com (accessed on 5 August 2020). Two firmness readings (N) were taken using a handheld penetrometer with an 11 mm diameter tip (Effegi pressure tester, Facchini, Alfonsine, Italy) on the opposite sides of each fruit. Two TSS (°Brix) readings were measured using a prism refractometer (Programable refractometer, 300037, SPER Scientific Ltd., Scottsdale, AZ, USA) from freshly juiced apples. 

### 4.4. Measurement of Plant Hormones 

#### 4.4.1. Ethylene 

Eight randomly selected fruit from each treatment were repeatedly used for the ethylene measurement. Before each measurement, apples were taken out of the cold storage and left overnight to reach room temperature. Fruit was weighed and placed in 2 L glass bottles. Bottles were sealed for an hour with a lid containing a rubber port where a syringe was used to collect 1 mL of headspace gas after gently shaking the bottles to mix up the air inside. The gas sample was immediately injected into an SRI-8610c gas chromatograph equipped with a 0.5 mL sample loop. The samples were separated by a capillary column (15 m × 0.32 mm Restek Rt-SPLOTTM, Chromatographic Specialties Inc., Brockville, ON, Canada). The ethylene was detected using a flame ionization detector and the readings were obtained at ppb (Varian Inc., Mississauga, ON, Canada). Pure ethylene (5 ppm) was used as the standard (BOG Gases, Mississauga, ON, Canada).

#### 4.4.2. Phytohormones and Metabolites

Three randomly selected fruit from each treatment were flash-frozen in liquid nitrogen and kept at −80 °C for the hormone analysis. Each about 25 mg of freeze-dried powdered sample (Three technical replicates per biological replicate) were homogenated with methanol-formic acid- Milli-Q H_2_O (5:1:4) solution and kept at −20 °C for an hour (methanol double extraction method). The supernatant was then collected by centrifugation (15 min, 14,000 rpm) and dried using nitrogen gas in a fume hood. The dried samples were reconstituted using a buffer solution (0.1% formic acid:acetonitrile = 97:3). The supernatant was then transferred to a 96-well collection plate. Metabolites were separated by reverse-phase ultra-performance liquid chromatography (UPLC) system connected with a mass spectrometer (MS) (Waters, Mississauga, Canada) by injecting a five μL aliquot of sample onto an Acquity B.E.H. Column (2.1 × 50 mm, i.d. 2.1 mm, 1.7 μm). Single ion recording mode was used to measure the metabolites. A standard curve was used for the quantification as described by Erland et al. [59]. 

### 4.5. Phospholipase-D Assay

Three randomly selected fruit from each treatment were used for the phospholipase D assays. A PLD assay kit was used to analyze based on the manufacturer’s recommended protocol (Cat. No. MAK137, Sigma-Aldrich 3050, St. Louis, MO 63103, USA). Briefly, all reagents were equilibrated to room temperature before use. In this case, 10 microliter of homogenate samples and standard solutions were separately added to the 96 well flat-bottom plates. Then the Master Mix was quickly added to each well and mixed thoroughly using a horizontal shaker (Biotek, Nepean, ON, Canada). The reaction was incubated at room temperature for 10 min, and the initial measurements were taken at 570 nm (A_570_)_initial_. After the first measurement, the plate was incubated for another 20 min, and then final measurements were taken (A_570_)_final_. The below equation was used to calculate the PLD activity of the sample (One unit of PLD catalyzes the formation of 1 µmole choline per minute at pH 7.4).

$$\mathrm{PLD}\;\mathrm{activity}\;\left(\mathrm{units}/L\right)=\frac{\left(A570)\mathrm{final}-\left(A570\right)\mathrm{initial}\right)}{\mathrm{Slope}\;\mathrm{of}\;\mathrm{the}\;\mathrm{standard}\;\mathrm{curve}\;\times t}\;\times \;\mathrm{dilution}\;\mathrm{factor}$$

### 4.6. Bitter Pit (BP) Assessment

In this case, 14 fruit per replicate per treatment were continuously observed for BP development. Incidence of the BP was assessed based on the presence or absence of BP signs on the fruit (for example, lesions with light to dark or deep color surrounded in the calyx end or any localized area of the fruit). Progression of the BP was calculated based on the difference in the incidence of BP between 0 days and 120 days postharvest. 

### 4.7. Gene Expression Analysis

Quantitative reverse transcription PCR (qPCR) was conducted for six genes representing phospholipase D enzyme and calcium sensor proteins. One microgram of total RNA extracted from fruit samples was reverse transcribed with Superscript II reverse transcriptase (Invitrogen, Burlington, ON, Canada). qPCR reactions were performed in 20 μL, containing 10 μL SYBRTM Green (Fisher Scientific, Mississauga, ON, Canada), two μL of cDNA and one μL of 400 nM of forward and reverse primers (Table S1) and seven μL of nuclease-free water. Three biological and technical replicates for each gene were analyzed using a CFX96 Real-Time PCR detection system (BioRad, Mississauga, ON, Canada). *Malus domestica Actin* (*MdACT*) and *Histone-3* (*MdHIS-3*) genes were used as reference genes to normalize the gene expression of a target gene. The gene expression was quantified using the 2^−ΔΔCt^ method [60]. 

### 4.8. Statistical Analysis

The experiment was conducted as randomized complete block design comprising three treatments with four replicates. Data collected for fruit quality and gene expression studies were analyzed using a repeated measured ANOVA with general linear mixed models (proc GLIMMIX) in SAS v9.4 (SAS Institute, Raleigh, NC, USA). An F test was used to test the equality of the variance of the fixed effects. The fixed effect variance was partitioned into fixed effects of treatment, day, and their combination. The day was considered as a repeated measured sequence of the analysis. A compound symmetric (cs) covariance type was used for the analysis. Shapiro-Wilk normality tests and studentized residual plots were used to test error assumptions of variance analysis, including random, homogenous, and normal distribution of error. Means were calculated using the LSMEANS statement, and significant differences between the treatments were determined by the Tukey-Kramer test with α = 0.05 and are mentioned in each figure or table. 

## Figures and Tables

**Table 1:** Variations in fruit quality traits and phytohormones at harvest (commercial maturity).

Parameter		Treatments		
		Control	Hexanal	Harvista^TM^
Firmness (N)		57.07 ± 1.54 ^a^	60.05 ± 1.67 ^a^	59.09 ± 1.24 ^a^
TSS (°Brix)		13.23 ± 0.08 ^a^	13.51 ± 0.0.1 ^a^	13.57 ± 0.08 ^a^
Blush Color	a *	32.72 ± 0.24 ^a^	31.58 ± 0.48 ^a^	30.44 ± 0.35 ^a^
	b *	15.23 ± 0.08 ^a^	13.11 ± 0.20 ^a^	13.35 ± 0.27 ^a^
	Lightness (L)	32.01 ± 1.69 ^a^	35.41 ± 1.68 ^a^	37.95 ± 1.68 ^a^
	Chroma (C)	36.13 ± 1.86 ^a^	34.28 ± 1.85 ^a^	33.28 ± 1.86 ^a^
	Hue Angle (H)	24.98 ± 1.88 ^a^	23.11 ± 1.88 ^a^	23.76 ± 1.88 ^a^
Background Color	a *	−2.84 ± 0. 50 ^b^	08.33 ± 0.62 ^ab^	12.80 ± 0.80 ^a^
	B *	25.53 ± 0.21 ^a^	20.63 ± 0.21 ^a^	20.64 ± 0.39 ^a^
	Lightness (L)	59.79 ± 2.76 ^a^	55.05 ± 4.34 ^a^	52.25 ± 2.78 ^a^
	Chroma (C)	24.37 ± 1.80 ^a^	23.78 ± 1.73 ^a^	28.54 ± 1.34 ^a^
	Hue Angle (H)	95.22 ± 10.52 ^a^	69.12 ± 10.52 ^a^	62.96 ± 8.95 ^a^
Phytohormonesand metabolites	Ethylene (nL/kg/hr)	48.83 ± 1.38 ^a^	39.97 ± 2.29 ^ab^	36.00 ± 2.05 ^b^
	ABA (ng/g, DW)	737.73 ± 10.8 ^b^	603.47 ± 12.03 ^c^	968.41 ± 11.71 ^a^
	Zeatin (ng/g, DW)	423.49 ± 8.81 ^b^	650.91 ± 8.77 ^a^	735.02 ± 9.61 ^a^
	Melatonin (ng/g, DW)	164.19 ± 7.05 ^a^	128.87 ± 5.4 ^a^	135.50 ± 4.31 ^a^
	Tryptophan (ng/g, DW)	4496.46 ± 117 ^c^	12,964.0 ± 161 ^a^	9220.23 ± 90 ^b^

Each value of parameters such as color, firmness, total soluble solids (TSS) and ethylene represents the mean ± SE of eight fruit. Each value of phytohormones represents the mean ± SE of nine replicates (three fruit each with three technical replicates). Means with the different letters indicate significant differences among control, hexanal and Harvista^TM^ treatments based on the Tukey-Kramer test at α = 0.05 at harvest.

**Table 2:** Variation in firmness and TSS in ‘Honeycrisp’ apples throughout the storage.

Parameter	Treatment	Storage Time (Days)				
		0	30	60	90	120
Firmness (N)	Control	57.07 (1.54) ^a–c^	54.87 (2.46) ^a–d^	52.58 (1.01) ^a–d^	51.71 (1.01) ^cd^	47.38 (1.84) ^b-d^
	Hexanal	60.05 (1.67) ^ab^	57.15 (2.36) ^a–c^	54.93 (1.53) ^a–d^	52.73 (1.33) ^a–d^	52.05 (2.18) ^a–d^
	Harvista	59.09 (1.24) ^ab^	57.94 (1.65) ^a–c^	54.01 (1.82) ^a–d^	52.88 (1.33) ^a–d^	49.79 (1.01) ^cd^
TSS (°Brix)	Control	13.23 (0.08) ^a–c^	13.55 (0.16) ^ab^	12.89 (0.18) ^c^	12.93 (0.11) ^bc^	13.03 (0.15) ^a–c^
	Hexanal	13.51 (0.10) ^ab^	13.65 (0.11) ^a^	13.56 (0.19) ^ab^	13.60 (0.05) ^a^	13.40 (0.17) ^a–c^
	Harvista	13.57 (0.08) ^a^	13.44 (0.17) ^ab^	13.58 (0.19) ^a^	13.44 (0.09) ^ab^	13.45 (0.18) ^ab^

Each value represents the least-squares means ± SE of eight fruit. LS-means with the same letter are not significantly different when comparing treatments with days postharvest based on the Tukey-Kramer test at α = 0.05.

**Table 3:** Effects of preharvest sprays on fruit quality traits fresh weight, firmness and TSS in ‘Honeycrisp’ apples after removal from cold storage (2.5 °C) to room temperature storage (~20 °C).

		Removal after 30 d		Removal after 60 d		Removal after 90 d	
Parameter	Treatment	7 Days	14 Days	7 Days	14 Days	7 Days	14 Days
Weight (g)	Control	261 ± 17.6 ^bc^	263 ± 17.6 ^bc^	259 ± 14.2 ^b^	254 ± 17.5 ^b^	343 ± 22.1 ^a^	334 ± 21.0 ^a^
	Hexanal	284 ± 17.8 ^a^	285 ± 8.0 ^a^	282 ± 18.0 ^a^	278 ± 18.0 ^a^	339 ± 28.79 ^a^	314 ± 55.45 ^ab^
	Harvista	258 ± 3.1 ^c^	261 ± 2.7 ^c^	257 ± 3.2 ^b^	252 ± 4.1 ^b^	279 ± 28.49 ^b^	266 ± 28.29 ^b^
Firmness (N)	Control	54.31 ± 2.55 ^c^	54.78 ± 3.87 ^b^	55.73 ± 3.83 ^b^	52.82 ± 4.91 ^a^	50.72 ± 4.04 ^a^	52.69 ± 4.51 ^a^
	Hexanal	59.8 ± 3.99 ^b^	60.82 ± 5.02 ^a^	58.79 ± 5.81 ^ab^	54.99 ± 2.09 ^a^	53.93 ± 2.96 ^a^	51.52 ± 3.87 ^a^
	Harvista	64.07 ± 2.8 ^a^	64.48 ± 1.38 ^a^	60.76 ± 2.42 ^a^	52.08 ± 7.4 ^a^	52.19 ± 3.32 ^a^	49.27 ± 4.99 ^a^
TSS (°Brix)	Control	12.15 ± 0.42 ^b^	12.03 ± 0.82 ^b^	12.30 ± 0.07 ^b^	12.48 ± 0.33 ^a^	12.35 ± 0.07 ^c^	12.82 ± 0.43 ^b^
	Hexanal	13.06 ± 0.13 ^a^	12.98 ± 0.07 ^a^	13.00 ± 0.11 ^a^	12.86 ± 0.09 ^a^	13.77 ± 0.24 ^a^	13.50 ± 0.5 ^a^
	Harvista	12.92 ± 0.18 ^a^	12.98 ± 0.26 ^a^	12.85 ± 0.21 ^a^	12.87 ± 1.56 ^a^	13.02 ± 0.49 ^b^	13.72 ± 0.76 ^a^

Fruit was removed from cold storage after 30, 60 and 90 days postharvest and kept for 14 days at room temperature at ~20 °C (. Values represent the mean ± SD of 5 randomly selected fruit. Means followed by different letters indicate significant differences among hexanal, Harvista^TM^ and control treatments at the same sampling time based on the Tukey-Kramer test at α = 0.05.

## Data Availability

All data supporting the findings of this study are available within the paper and within its Supplementary Materials published online.

## Supplementary Materials

The following are available online at www.mdpi.com/2223-7747/10/11/2332/s1, Table S1: Primer sequence of genes putatively encoding phospholipase D and calcium sensor proteins; Table S2: Effects of preharvest sprays on fruit quality traits in ‘Honeycrisp’ apple after removal from cold storage (2.5 °C) to room temperature storage (~20 °C); Figure S1: Effects of preharvest sprays on color parameters throughout the cold storage.
