# Supplementary material for: Preharvest Spray Hexanal Formulation Enhances Postharvest Quality in ‘Honeycrisp’ Apples by Regulating Phospholipase D and Calcium Sensor Proteins Genes
Source: Plants (Basel). 2021 Oct 28;10(11):2332. doi: 10.3390/plants10112332 (PMC8623384; doi:10.3390/plants10112332)
Supplement: Supplementary file 1 [file plants-10-02332-s001.zip › plants-1434556-supplementary.pdf]

Preharvest Spray Hexanal Formulation Enhances Postharvest Quality in 'Honeycrisp' Apples by Regulating Phospholipase D and Calcium Sensor Proteins Genes

Karthika Sriskantharajah [1], Walid El Kayal [2,3], Murali M. Ayyanath [1], Praveen K. Saxena [1], Alan J. Sullivan [1], Gopinadhan Paliyath [1] and Jayasankar Subramanian [2*]

Supplementary Materials

Table S1: Primer sequence of genes putatively encoding phospholipase D and calcium sensor proteins

No	Gene	Gene ID/accession # (*Malus domestica*)	Forward primer	Reverse Primer
1	MdCaM2	MDP0000183898	ATGGGACCATTGATTTTCCA	CAGCCTCACGAATCATCTCA
2	MdCaM4	MDP0000277474	TGCTACCTCCCATCCTTCAG	CATCTTCCTCGCCATCAAAT
3	MdCML1	MD02G1202000	GACGGTTACCTGGACCGTTA	TTCCCGTCTTTGTTGGAGTC
4	MdCML18	MD10G1094100	TGCAACCTCATCTTTTGCAG	TTCCCTCACAATCCCTTCAG
5	MdPLDα1	XM_008375733.3	CAGAGGTGGAGAAAGCAAGG	AGCTGCATCTTCAGGCGTAT
6	MdPLDα4	XM_008393094.3	TGAGATCCAAGGGGAGTTTG	GTTATCGCCATTGTCGGACT
7	MdAct	XM_008362405.3	GTGGATTGCAAAGGCAGAGT	CATAATTTGCTCGCCTCCAT
8	MdHis3	AY347801.1	TGGAACTGTTGCTCTTCGTG	CTCAAACAACCCGACAAGGT

Sequencing details to the gene set 1-6 were obtained from the Genomic Database for Rosaceae and 5 -8 from the NCBI database.

Table S2: Effects of preharvest sprays on fruit quality traits fresh weight, firmness and TSS in 'Honeycrisp' apples after removal from cold storage (2.5 ^{o}C) to room temperature storage (~20 ^{o}C)

		Days removal after cold storage		
**Variant**	**Treatment**	**30**	**60**	**90**
Mean fresh weight over 14 d at 20 ^{o}C (g)	Control	262 (252 - 272) ^{b}	257 (247 - 266) ^{b}	339 (317 - 360) ^{a}
	Hexanal	284 (275 - 294) ^{a}	280 (271 - 290) ^{a}	326 (305 - 348) ^{a}
	Harvista	260 (250 - 269) ^{b}	254 (245 - 264) ^{b}	272 (251 - 293) ^{b}
Mean firmness over 14 d at 20 ^{o}C (N)	Control	54.55 (52.88 - 56.21) ^{c}	54.28 (52.33 - 56.27) ^{a}	51.70 (49.66 - 53.74) ^{a}
	Hexanal	60.31 (58.64 - 61.98) ^{b}	56.9 (54.94 - 58.85) ^{a}	52.72 (50.68 - 54.77) ^{a}
	Harvista	64.27 (62.6 - 65.94) ^{a}	56.42 (54.47 - 58.37) ^{a}	50.73 (48.69 - 52.77) ^{a}
Mean TSS over 14 d at 20 ^{o}C (^{o}Brix)	Control	12.09 (11.87 - 12.31) ^{b}	12.89 (12.75 - 12.99) ^{a}	12.58 (12.32 - 12.84) ^{b}
	Hexanal	12.99 (12.77 - 13.22) ^{a}	12.93 (12.82 - 13.03) ^{a}	13.63 (13.37 - 13.89) ^{a}
	Harvista	12.95 (12.73 - 13.17) ^{a}	12.86 (12.75 - 12.97) ^{a}	13.36 (13.10 - 13.63) ^{a}

*Fruit were removed from cold storage after 30, 60 and 90 days postharvest and kept for 14 days at room temperature at ~20 ^{o}C (RmT). Values represent the mean ± SD of 10 randomly selected fruit. Means followed by different letters indicate significant differences among hexanal, Harvista^{TM} and control treatments at the same sampling time based on the Tukey-Kramer test at $\alpha = 0.05$.

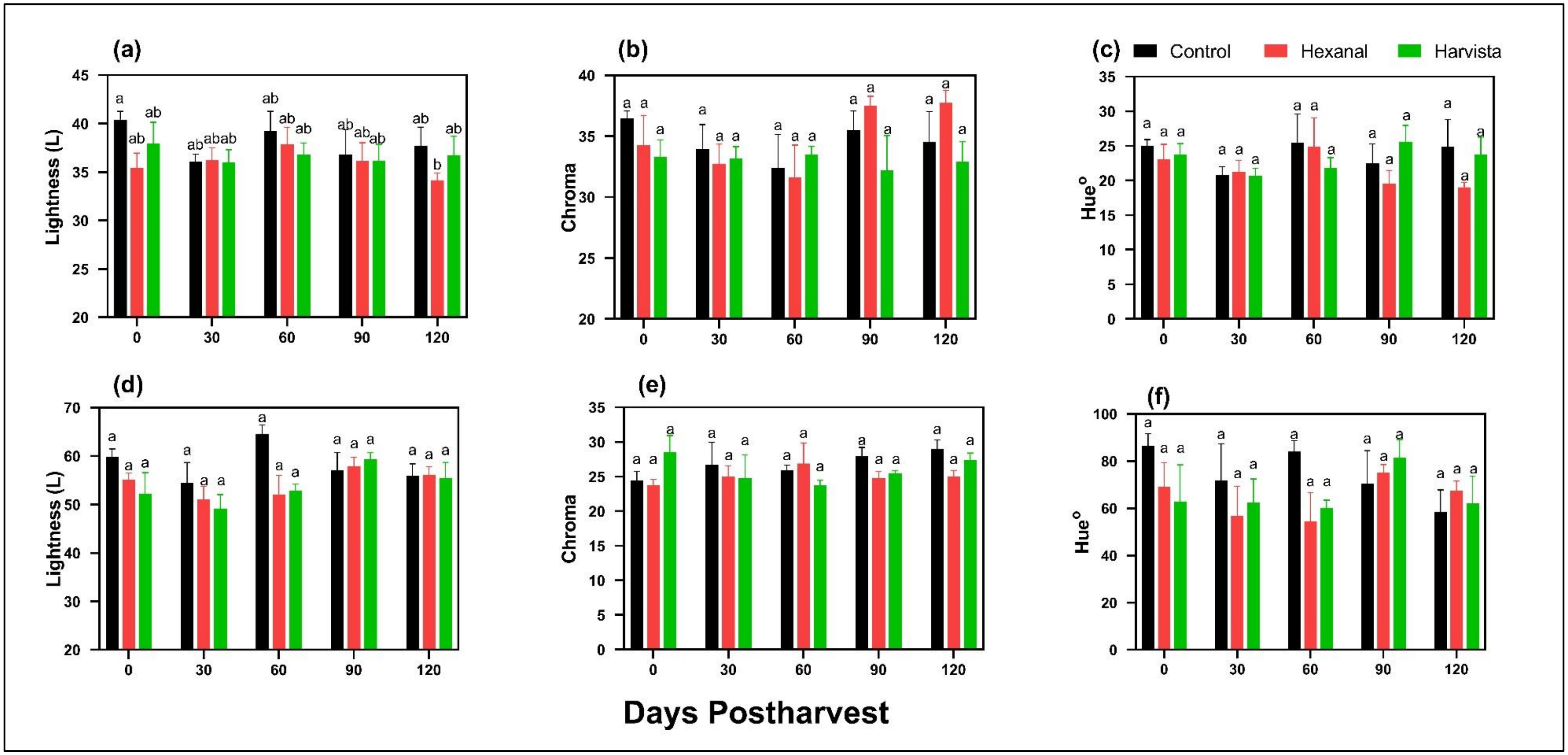

Figure S1: The effects of preharvest sprays on variations in color parameters (a-c) of blush and (d-f) of background of 'Honeycrisp' throughout the cold storage (2.5 °C). L indicates the brightness in the CIE lab system readings. Chroma and Hue angel were calculated using the software available at at http://www.easyrgb.com (accessed 5, August 2020). Each value represents the least-squares means ± SE of eight fruit. LS means with the same letter are not significantly different when comparing treatments with days postharvest based on the Tukey-Kramer test at $\alpha = 0.05$.
